# Stem cell-derived exosomes: a potential therapeutic strategy for enhancing tendon stem/progenitor cells function in tendon-bone healing

**DOI:** 10.1186/s13018-025-06060-z

**Published:** 2025-07-15

**Authors:** Junjie Tang, Peng Shen, Xinyuan Wu, Minhao Chen, Hua Xu

**Affiliations:** Department of Orthopaedics, Medical School of Nantong University, Affiliated Hospital of Nantong University, Nantong, 226001 China

**Keywords:** Exosomes, Tendon stem/progenitor cells, Tendon-bone healing, Tendon-bone insertion injures

## Abstract

Tendon-bone insertion (TBI) injuries and diseases are one of the common musculoskeletal conditions that can severely impair an individual’s daily activities and quality of life. The healing process following an injury is intricate and depending on microenvironmental factors such as mechanical loading, inflammatory responses, and the extracellular matrix. Tendon stem/progenitor cells (TSPCs) primarily contribute to the replenishment of tendon cells via self-renewal and differentiation, which is essential for tendon-bone healing. Exosomes, small extracellular vesicles from various cell types, have attracted significant interest for their potential in regenerative medicine, particularly for treating tendon disorders. Recent studies indicate that exosomes from various cell sources, including mesenchymal stem cells (MSCs) and adipose-derived stem cells (ADSCs), can effectively modulating the activity of TSPCs and enhance their therapeutic potential. This review provides an overview of the mechanisms and functions of stem cell-derived exosomes in altering the properties of TSPCs and facilitating tendon-bone healing. In conclusion, exosomes offer a promising therapeutic approach for TBI injuries. However, further clinical validation is required. Utilizing the regenerative capabilities of exosomes could address promotion of tendon-bone healing and enhance the quality of life for affected patients.

## Introduction

Tendon-bone insertion (TBI) injuries, including anterior cruciate ligament (ACL) and rotator cuff (RC) injuries and so on, frequently affect patients’ work and daily activities, particularly during sports and recreational pursuits [1]. Such injuries are prevalent and present considerable difficulties for the musculoskeletal system because of the distinctive characteristics of the tendon-bone interface, which plays a vital role in the transfer of forces from muscles to bones. The repercussions of these injuries are not limited to short-term pain and distress; they may result in chronic impairments and diminished life quality if they are not effectively addressed [2]. This is where the importance of tendon-bone healing comes into focus.

Tendon-bone healing is a multifaceted and evolving procedure influenced by a variety of elements. This sophisticated recovery process involves four distinct stages: the inflammatory phase, the proliferative phase, the remodeling phase, and the maturation phase [3, 4]. Over the past few years, a substantial amount of data has been gathered from animal models and clinical trials, highlighting the exceptional regenerative potential of stem cells [5–7]. Among the various stem cell types, Tendon stem/progenitor cells (TSPCs), which are multipotent stem cells, are mainly accountable for replenishing tendon cells through self-renewal and differentiation, playing a pivotal role in tendon regeneration. Alongside tenocytes, which maintain tendon homeostasis under normal conditions, TSPCs constitute the two major cell types found in tendons. These important cells were first identified in humans and mice in 2007, marking a significant milestone in the field of tendon biology and repair [6, 8]. During tendon repair, regeneration, and homeostasis maintenance, TSPCs demonstrate the expression of genes associated with tendon lineage. They are crucial because of their ability to become osteoblasts that create bone and chondrocytes that produce cartilage. The decline of TSPCs’ function may result in tendon issues associated with acute trauma and can diminish the tendon’s ability to heal and regenerate in humans [6, 9]. This decline in regenerative potential underscores the importance of understanding cellular mechanisms at the molecular level, which brings us to a pivotal discovery in cellular biology.

In 1983, two studies, one in JCB and the other in Cell, independently revealed that transferrin receptors on reticulocytes engage with approximately 50 nm functional vesicles [10, 11]. These vesicles are derived from developing sheep reticulocytes and are secreted into the extracellular space [5]. Exosomes are small membrane-bound vesicles, measuring between 40 and 160 nm in size, released by a variety of cells. They are filled with a diverse array of substances, such as nucleic acids (DNA, mRNA, and noncoding RNA), lipids, and a multitude of proteins [12]. Harnessing their rich composition, stem cell-derived exosomes have emerged as a cutting-edge therapeutic strategy for healing and regeneration in medical applications. They effectively overcome the limitations of traditional TSPCs-based therapies, such as cell heterogeneity, instability, and delivery challenges, and provide a compelling rationale for using stem cell-derived exosomes as a functional enhancement strategy for tendon-bone healing [5]. These nanoscale vesicles facilitate intercellular communication and play crucial roles in the regulation of physiological and pathological processes [12]. Exosomes offer several benefits compared to conventional stem cell treatments, such as their low risk of immune reactions, minimal infusion-related side effects, simplicity in terms of acquisition and storage, and the absence of cancer-causing risks and ethical dilemmas [13, 14].

Exosomes are capable of passing on the curative properties of their originating cells, including mesenchymal stem cells (MSCs) and adipose-derived stem cells (ADSCs), by directly transferring their pluripotent or multipotent capabilities [15, 16]. Research has shown that exosomes can promote cell proliferation, migration, and immune regulation, making them capable of treating a variety of diseases in orthopedic surgery, neurosurgery, plastic surgery, and other fields [17]. Jin’s research indicates that exosomes derived from stem cells of human shed primary teeth (SHED-Exos) are rich in potent anti-aging messages. These youthful bio-nanoparticles are able to reduce signs of aging in mature TSPCs and maintain their ability to form tendon tissue [18]. Umbilical cord-derived mesenchymal stem cells (UC-MSCs), easily extracted from umbilical cord Wharton’s jelly, are a promising source of exosomes, offering an ethically sound and accessible option [19]. Hu investigated the impact of exosomes from ADSCs on TSPCs’ migratory capacity, emphasizing the importance of stem cell homing and migration for tissue repair [20]. The healing properties of exosomes originating from stem cells are realized through responses that are tailored to specific tissues and molecular signaling pathways that target particular cell types. This review renders a synoptic view of the mechanisms and functions of exosomes in impacting the performance of TSPCs and enhancing tendon-bone healing, underscoring their surgical applications and potential as a powerful substitute for conventional stem cell treatments.

## Therapeutic potential of exosomes in tendon-bone healing

**Fig. 1:**
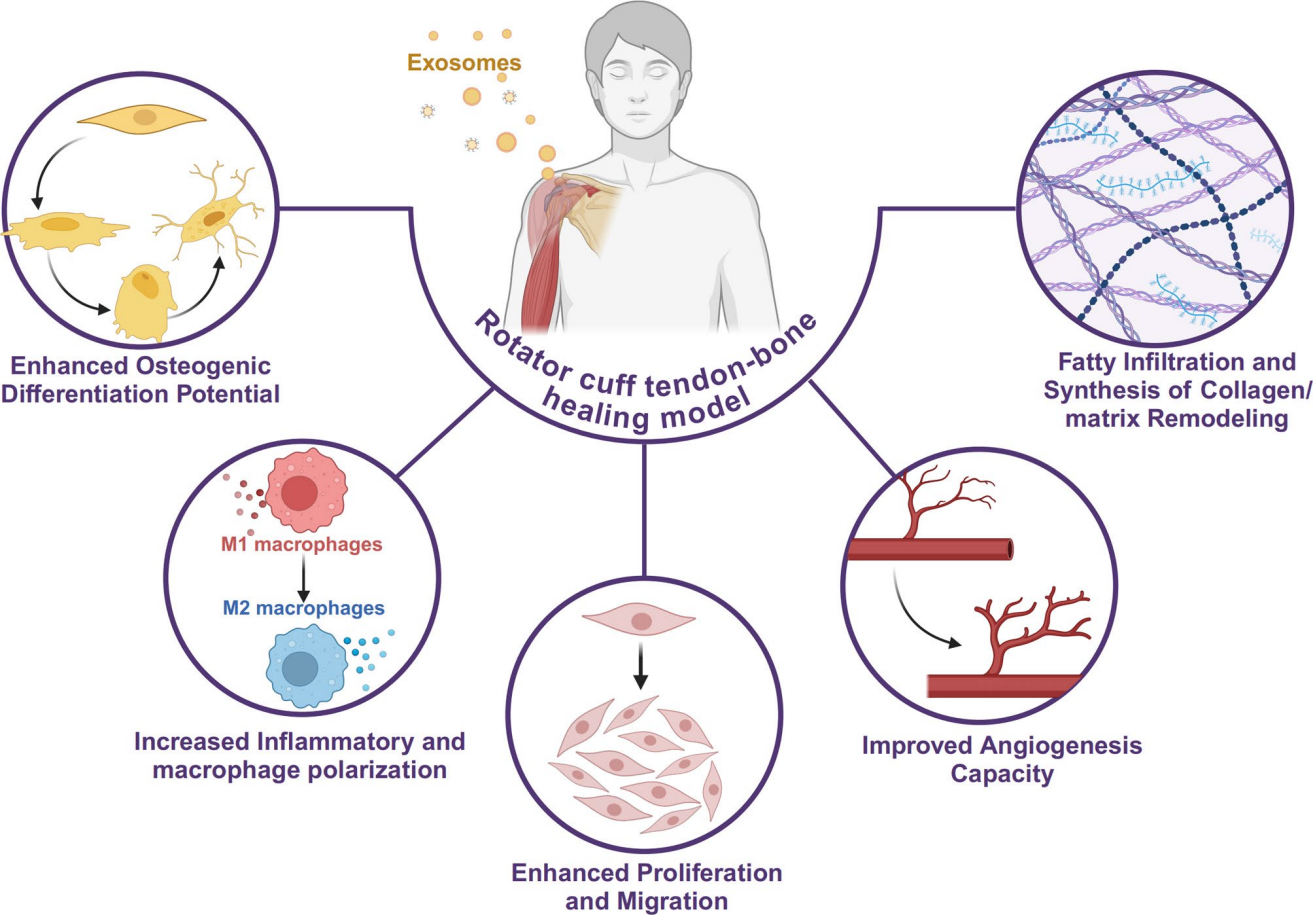
The rejuvenating effects of exosomes on tendon stem/progenitor cells include osteogenic differentiation potential, inflammatory and macrophage polarization, proliferative and migration, angiogenesis capacity, fatty infiltration and synthesis of collagen/matrix remodeling

The rejuvenating effects of exosomes on TSPCs are reflected in the restoration of their key functional attributes, including osteogenic differentiation potential, inflammatory and macrophage polarization, proliferative and migration, angiogenesis capacity, fatty infiltration and synthesis of collagen/matrix remodeling. (Fig. 1)

### Enhanced TSPCs tenogenic differentiation potential

One of the key hallmarks of TBI injuries is the increased ossification and calcification of the tendon tissue, leading to decreased flexibility and impaired mechanical properties. Exosomes enhance bone metabolism factor expression, thereby stimulating osteogenesis and suppressing osteolysis. Numerous investigations have indicated that bone resorption or osteolysis reduces the stiffness and strength at the tendon-bone junction, impeding the healing process of the TBI [21, 22]. Salhotra et al. reported that increased overall bone resorption and weaker bones attributable to osteoclast activity surpasses osteoblast during the ageing process. Chen et al. have demonstrated that exosomes originating from human umbilical vein endothelial cells (HUVECs) stimulate the osteogenic differentiation and migration of bone mesenchymal stem cells (BMSCs) [23]. Exosomes enriched with higher levels of miR-181b trigger the PRKCD/AKT signaling pathway, thereby promoting osteogenic differentiation in vitro and osteointegration in vivo [24]. Exosomes of miR29a-loaded BMSCs have the robust capacity to promote angiogenesis and osteogenesis in mice [25]. Therefore, promoting osteogenesis and inhibiting osteolysis can help tendon-bone healing.

Regenerating the TBI necessitates the coordinated differentiation of TSPCs towards the osteogenic lineage, alongside fibrocartilage formation. Effective tendon-bone integration is essential for restoring the enthesis’s complex biomechanical properties. Exosomes are rich in osteogenic signaling molecules, including bone morphogenetic proteins, Wnt proteins, and transcription factors like Runx2, which promote the osteogenic differentiation of stem cells. Han reported significant upregulation of tendon regeneration and cartilage differentiation-related protein expressions with polyaspartic acid polylactic acid-glycolic acid (PASP-PLGA) microcapsule exosome treatment [26]. The primary aim of TSPCs-based therapies for tendon repair is to induce the differentiation of these cells into the tenogenic lineage, resulting in the development of functional tendon-like tissue. Exosomes contain key tenogenic signaling molecules and transcription factors, including scleraxis, tenomodulin, and collagen type I (Col1), which are vital for tendon development and homeostasis [27]. When TSPCs are exposed to these exosomes, they show elevated expression of tenogenic markers and increased production of tendon-specific extracellular matrix components like collagen and proteoglycans. Lu’s research showed that platelets enriched with recombinant Yap1 protein (PLT-Exo-Yap1) are capable of suppressing cellular senescence, enhancing the stem-like properties of TSPCs, and boosting their potential for tenogenic differentiation [28]. Incorporating stem cell-derived exosomes into 3D tissue engineering constructs, like hydrogels or decellularized tendon matrices, significantly enhances TSPCs tenogenic differentiation and tendon-like tissue formation both in vitro and in vivo [29]. In summary, exosomes rich in osteogenic and tenogenic signaling molecules and transcription factors effectively promote the differentiation of TSPCs into the osteogenic and tenogenic lineages, enhance tendon-bone integration, and improve the quality of regenerated tendon tissue through various mechanisms and applications in tissue engineering constructs.

### Increased inflammatory and macrophage polarization

Macrophages are essential in the wound healing process, aiding in the positive aspects of inflammation and managing a variety of related processes. Throughout the wound healing process, these cells display distinct phenotypes that are instrumental in facilitating recovery. The M1 macrophage phenotype is defined by its phagocytic and pro-inflammatory activities, while the M2 phenotype is associated with the resolution of inflammation and tissue repair [30]. The researchers found that TSPCs from chronically torn RC tendons exhibit a positive feedback loop with macrophages, promoting polarization towards the M1 phenotype, which is associated with pro-inflammatory responses and tissue degeneration. This cross-talk impairs their multipotency, hindering the healing process [31].

Relevant research has indicated that exosomes possess the capacity to regulate macrophage polarization thereby slowing down the inflammatory response and facilitating tendon-bone healing. BMSC-Exos have been demonstrated to shift the polarization of M1 to M2 macrophages through miR-23a-3p, which lessens initial inflammation at the TBI and supports early healing following ACL reconstruction [26, 32]. Additionally, BMSC-Exos are capable of suppressing the polarization of M1 macrophages and the release of their pro-inflammatory cytokines [33, 34]. Chen et al. described that conditioned medium of human BMSC influenced macrophage polarization via the Smad2/3 signaling pathway and promoted tendon-bone healing in a rat model of rotator cuff repair [35, 36]. Xu et al. revealed that exosomes from MSCs accelerated the healing of the tendon-bone junction by influencing the polarization of macrophages in rats models [37]. Rong et al. reported that an extracellular vesicles-cloaked enzymatic nanohybrid can boost the activity of M2 macrophages in the healing tendons of animal models, which has been linked to a reduction in scar tissue, faster recovery, lower levels of inflammation, and better mechanical properties [38]. In conclusion, exosomes can disrupt the positive feedback cycle by redirecting macrophage polarization from the M1 phenotype towards the M2 phenotype, which is anti-inflammatory and promotes tissue regeneration. It is worth noting that although this section discusses how exosomes can alleviate tendon-bone injuries by influencing macrophage polarization, whether TSPCs are involved needs to be further substantiated in future research.

### Enhanced proliferation and migration

Exosomes have been shown to stimulate the proliferation of TSPCs, thereby expanding this critical cell population for tissue regeneration, and also enhance their migratory and homing abilities, which facilitates their recruitment to the site of tendon injury and integration into the damaged tissue. Ren et al. have discovered that exosome extracts can stimulate cell growth and movement by increasing the expression of tendon-specific markers in a co-culture system in vitro [5, 39]. Li et al. investigated that exosomes from BMSCs, enriched with TGF-β1,enhanced the proliferation, migration, and fibrogenic activity of tenocytes in the RC [18, 40]. Song et al. investigated exosomal miR-144-3p delivery enhances tenocyte proliferation and migration by targeting AT-rich interactive domain 1A [41]. Yu et al. revealed that MSCs-derived exosomes enhanced proliferation, migration and tenogenic differentiation of TSPCs during tendon repair [42]. These findings highlight the potential of stem cell-derived exosomes to enhance TSPCs proliferative and migratory abilities, thereby improving regenerative potential.

### Improved angiogenesis capacity

Angiogenesis, the formation of new blood vessels, is essential for tendon repair and regeneration by supplying oxygen and nutrients to the injured tissue. Vascularization and bone regeneration are interrelated processes in bone reconstruction [43, 44]. However, in many cases, the inherent angiogenic capacity of TSPCs may be limited, hindering their ability to effectively promote vascularization and, consequently, the overall healing process. Exosomes boost the production of collagen and the formation of new blood vessels by increasing mRNA levels and secreting factors that promote angiogenesis, as well as regulatory proteins [45]. With strong pro-angiogenic capabilities, these vesicles effectively enhance the proliferation and tube formation capacity of endothelial cells, processes that are crucial to the development of new blood vessels [46].

Contemporary research has reported that this enhancement of angiogenesis is believed to be mediated by the specific cargo carried within the exosomes, which can stimulate and support the various stages of the angiogenic process. These exosomes are notably abundant in vascular endothelial growth factor (VEGF), a principal catalyst for angiogenesis. The delivery of VEGF and other pro-angiogenic factors to endothelial cells significantly enhances their proliferation, migration, and tube formation, which are critical steps in the development of new blood vessels [47]. By facilitating these processes, stem cell-derived exosomes provide a robust support system for the construction and repair of new vascular networks [48].

Interestingly, the angiogenic potential of exosomes can be further enhanced through various strategies, such as the manipulation of the exosome cargo or the culture conditions of the parent TSPCs. For instance, Wu et al. found that low doses of Fe_3_O_4_ nanoparticles combined with static magnetic field may stimulate exosomes to promote angiogenesis and accelerate bone defect healing in vitro and in vivo critical-sized calvarial defect rat model [49]. Liu et al. indicated that Hypoxia-Exos administration significantly enhanced angiogenesis, cell proliferation, and migration through exosomal miR-126 in vitro experiments and in vivo bone fracture model [50]. Furthermore, Zhang et al. described that Hypo-Exos delivered via an adhesive hydrogel demonstrate angiogenic effects in the peri-graft area following ACL reconstruction, potentially enhancing peri-graft bone formation and remodeling [51]. In summary, the enhancement of angiogenesis through the use of exosomes represents a promising strategy for improving the regenerative capacity of TSPCs.

### Fatty infiltration and synthesis of collagen/matrix remodeling

As injuries occurs, TSPCs increasingly differentiate towards the adipogenic lineage, leading to the accumulation of fat within the tendon tissue, a phenomenon known as fatty infiltration or tendinosis [52]. Studies have demonstrated that cells contain elevated levels of specific miRNAs, such as miR-335, which target key regulators of adipogenesis. MiR-335 directly targets the transcription factor Runx2, a key regulator of osteogenesis, and promotes the expression of adipogenic transcription factors such as PPARγ [53, 54].

In contrast, exosomes derived from stem cells have been demonstrated to exert an opposing effect, promoting the maintenance of the tenogenic phenotype and inhibiting the adipogenic differentiation of TSPCs [55]. Wang et al. discuss that ADSCs-derived exosomes were found to prevent fatty infiltration, enhance tendon-bone healing, and improve biomechanical properties in chronic RC during repair [5, 52]. These exosomes contain a different cargo of miRNAs and other bioactive molecules that can counteract the pro-adipogenic signals, thus facilitating tendon-bone healing.

Stem cell-derived exosomes are gaining recognition for their therapeutic potential in tendon-bone healing, particularly in enhancing osteogenesis, modulating inflammation, and promoting cell proliferation and migration. They also play a role in regulating collagen synthesis and matrix remodeling, which are essential for maintaining tendon and bone integrity. Beyond the previously discussed components, exosomes contribute to the healing of tendon-bone junctions by enhancing the integrity of the blood-spinal cord barrier, safeguarding and regenerating neural tissues, modulating immune responses, and strengthening biomechanical properties. In summary, exosomes offer a multifaceted approach to tendon-bone healing by targeting key aspects of tissue repair and regeneration, establishing them as a potential therapeutic approach for TBI injuries.

## Potential mechanisms

**Fig. 2:**
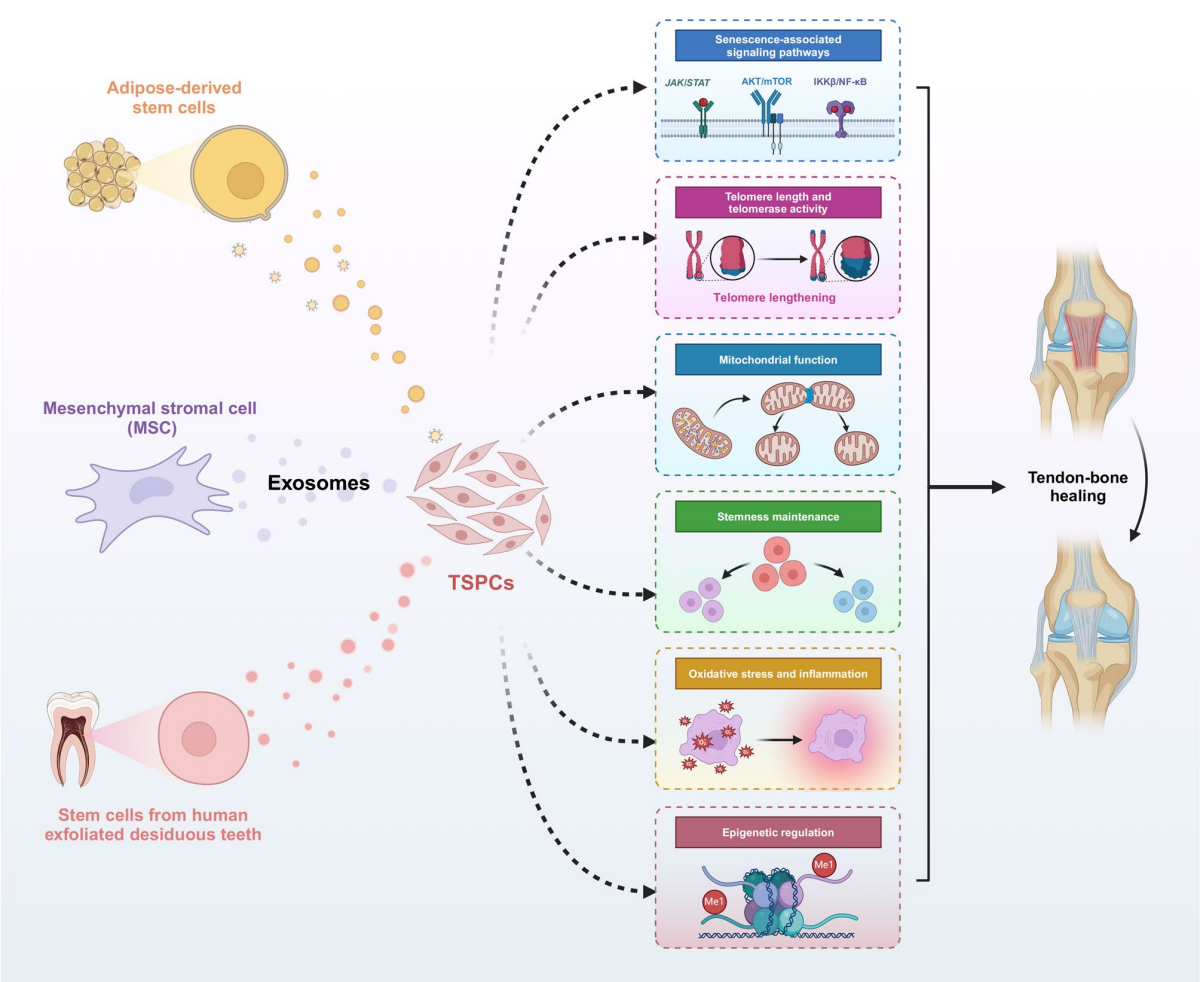
The mechanisms by which stem cell-derived exosomes act on tendon stem/progenitor cells to promote tendon-bone healing include impaired signaling pathways, telomere shortening, mitochondrial dysfunction, loss of stemness, increased oxidative stress, and dysregulated epigenetics

TBI injuries and age-related degeneration significantly impair function and quality of life. TSPCs are essential for tissue maintenance, but their regenerative capacity diminishes over time, leading to challenges in tissue repair and regeneration. This phenotype is marked by cellular and molecular changes such as impaired signaling pathways, telomere shortening, mitochondrial dysfunction, loss of stemness, increased oxidative stress, and dysregulated epigenetics. The mechanism by which stem cell-derived exosomes alter the properties of TSPCs is by affecting the aforementioned six aspects to exert their function. (Fig. 2)

### Modulation of senescence-associated signaling pathways

In the context of tendon injury and degeneration, studies have shown that exosomes can induce premature senescence in neighboring TSPCs through the transmission of senescence-associated molecular signals. Exosomes downregulate senescence-associated markers like p16^INK4a^ and p21^Waf1/Cip1^ by activating pro-proliferative and anti-apoptotic signaling pathways, including PI3K/Akt and MAPK/ERK [56, 57].

Stem cell-derived exosomes have been shown to positively influence the signaling pathways that are often dysregulated in senescent TSPCs. One key player in TSPCs senescence is the p16^INK4a^/Rb pathway, a well-characterized tumor suppressor mechanism that becomes activated during cellular aging. Senescent cells secrete exosomes enriched with p16^INK4a^, which can be internalized by TSPCs, leading to the upregulation of p16^INK4a^ and the subsequent induction of cell cycle arrest and senescence [58]. Lyu et al. propose that TGF-β/Smad is a key pathway regulating both damage-induced and developmentally programmed senescence. Exosomes also influence the Wnt/β-catenin signaling pathway, crucial for maintaining TSPCs stemness and promoting tenogenic differentiation. Exosomes derived from youthful or revitalized MSCs have demonstrated the ability to increase the expression of Wnt-related genes in aging TSPCs, thus rejuvenating their abilities for self-renewal and differentiation [21, 59].

A PubMed search for studies on senescent TSPCs was conducted on January 5, 2019, and updated on May 7, 2025, using the keywords: ((tendon) AND (ageing OR aging OR age OR senescence) AND (stem OR progenitor) cell. No language or publication year restrictions were applied. After scrutinizing the titles and summaries of the primary research articles on factors affecting senescent TSPCs, 116 articles were identified. This review comprises 24 studies, all on influencing factors of elderly TSPCs. Table 1 presents a comprehensive overview of the various factors that influence the aging of TSPCs across different animal models, including rats, mice, and humans. These factors range from extracellular vesicles like SHED-Exos, platelet-derived exosomes and circPVT1-exo which modulate histone methylation and suppress nuclear factor-κB to counteract aging, to signaling pathways such as noncanonical Wnt5a, JAK-STAT, AMPK/Nrf2/GPX4 and SIRT1/NF-κB, which play crucial roles in regulating senescence [18, 60–64]. Other factors include connective tissue growth factor (CTGF), aquaporin-1 (AQP1), glutamine, prim-O-glucosylcimifugin (POG), thrombospondin 1, dasatinib and quercetin which have been shown to reduce expression levels or protect TSPCs from aging-related deterioration [65–70]. Circular RNA PVT1 in humans has been identified for its role in sponging microRNA-199a-5p, while metformin’s effect on the AMPK/mTOR pathway delays tendon aging in rats [9, 71, 72]. Additionally, celecoxib, VEGF and rapamycin have been studied for their effects on aging-related markers, adipogenic differentiation, and autophagy, respectively [47, 73, 74]. The table also highlights the role of signaling pathways like IKKb/NF-κB in tendinopathy healing and the impact of Botox injections on cellular function of TSPCs [75]. Table 1 summarises the studies on factors of influence of TSPCs.

**Table 1:** Influencing factors of elderly TSPCs

Animal model	Factors	Mechanisms	References
Rats, mice	SHED-Exos	SHED-Exos modulate histone methylation and suppress nuclear factor-κB, thereby counteracting the aging of AT-SCs	[76]
Mice	Noncanonical Wnt5a	The JAK–STAT signaling cascade was triggered in senescent TSPCs	[60]
Rats	Connective tissue growth factor (CTGF)	The expression of CTGF was markedly reduced at both the protein and mRNA levels	[65]
Rats	AQP1	Elevated levels of AQP1 suppressed the activation of the JAK-STAT signaling pathway	[66]
Rats	Prim-O-glucosylcimifugin (POG)	POG protects TSPCs from deterioration associated with both passaging-induced and natural aging by simultaneously suppressing nuclear factor-κB, diminishing mTOR signaling, and enhancing autophagy.	[67]
Human	Circular RNA PVT1	Sponging microRNA-199a-5p	[77]
Rats	AMPK/mTOR	Metformin administration delays the aging process of tendons and boosts AMPK phosphorylation, while simultaneously decreasing mTOR phosphorylation	[71]
Rats	HPF1	PARP1-mediated poly-ADP ribosylation of HuR	[72]
Human	Celecoxib	Assaying the expression of aging-related markers	[73]
Rats	VEGF	Inhibit adipogenic differentiation	[47]
Rats	IKKb/NF-κB signaling	-	[75]
Rats	Rapamycin	Upregulating autophagy	[74]
Rats	JAK-STAT signaling	Tendon regeneration	[62]
Mice	Botox	Analysis of the signaling pathway indicated that Botox injections into the quadriceps muscle leads to PTEN/AKT-driven cellular aging of tendon-derived stem cells TSPCs	[78]
Rats	Extracellular Vesicle-Contained Thrombospondin 1	TSPC-EVs deliver THBS1 to inhibit the PI3K/AKT pathway	[68]
Rats	Platelet-derived exosomes	Exosomes regulate AMPK/Nrf2/GPX4 signaling and improve tendon-bone junction regeneration	[63]
Human	Glutamine	Ameliorating age-related osteoporosis	[69]
Human	Dasatinib and quercetin	Dasatinib and quercetin increases genetic expression of COL1A1 In vitro	[70]
Human	CircPVT1-exo	Modulating SIRT1/NF-κB pathway	[64]

### Restoration telomerase activity

Telomeres, the protective chromosome caps, shorten with each cell division, causing cellular senescence. To combat this issue, telomerase, a complex of RNA and proteins, is crucial for preserving the length of telomeres, which in turn safeguards the differentiation and self-renewal potential of progenitor cells [79]. In senescent TSPCs, telomere shortening and decreased telomerase activity contribute to their impaired regenerative potential [80]. Stem cell-derived exosomes enhance telomerase activity and lengthen telomeres in senescent TSPCs, reversing cellular aging markers.

The mechanism by which exosome-mediated cargo transfer can remodel telomere length and telomerase activity involves several steps. TSPCs internalize exosomes from youthful, robust cells through processes such as clathrin-dependent endocytosis, caveolae-dependent endocytosis, and macropinocytosis. Once internalized, the exosomes’ cargo is released into the cytoplasm of the recipient senescent TSPCs. The exosome-transferred telomerase reverse transcriptase (TERT) mRNA is translated into the catalytic subunit of telomerase, which combines with TERT to form the active telomerase complex [81]. This increased telomerase activity in senescent TSPCs enables the elongation of their shortened telomeres, effectively reversing the telomere attrition associated with cellular aging. Furthermore, miR-29b and miR-335 have been found to upregulate the expression of other telomerase-associated genes, such as dyskerin and NOP10, further potentiating telomerase function in TSPCs [82].

### Improvement of mitochondrial function

Mitochondrial dysfunction is a central factor in the function-related decline of TSPCs. These organelles are crucial for energy production, redox balance, and the signaling pathways that regulate TSPCs proliferation, differentiation, and tissue regeneration [83]. With the functional decline of TSPCs, their mitochondria experience changes in structure and function, resulting in reduced ATP production, and heightened oxidative stress [84]. Fortunately, exosomes have shown the potential to ameliorate mitochondrial function in these cells, which is vital for sustaining cellular energy homeostasis.

Current research has shown that exosomes can transfer mitochondrial components, including mitochondrial DNA (mtDNA), mitochondrial transcription factors, and even intact mitochondria, to target cells, thereby influencing their mitochondrial function and overall cellular homeostasis [85]. Exosomes benefit TSPCs by transferring functional mitochondria or their components. Besides directly conveying mitochondrial parts, exosomes can also modulate mitochondrial activity in TSPCs by transferring particular regulatory molecules like transcription factors, microRNAs, and signaling proteins. For instance, exosomes rich in the transcription factor PGC-1α, a principal controller of mitochondrial biogenesis, can boost mitochondrial gene expression, performance, and ATP synthesis in TSPCs. Exosome-encapsulated microRNAs, like miR-335, targeting negative regulators of mitochondrial function, have been demonstrated to enhance mitochondrial respiration and decrease oxidative stress in TSPCs [82]. This restoration enhances the organizational structure of the mitochondrial network and bolsters the respiratory capacity within TSPCs.

### Promotion of stemness maintenance

Maintaining the stem-like properties of TSPCs amidst cellular stress is essential for the conservation and restoration of tendon tissue [86]. Exosomes have been shown to preserve the multipotent differentiation capacity of TSPCs, maintaining their stem cell-like properties. This is mediated by the modulation of transcription factors and epigenetic regulators that are crucial for the maintenance of stemness. Research suggests that stem cell-derived exosomes expressing effectors like Wnt, Hedgehog, and β-catenin may contribute to maintaining stemness [87]. Hong et al. reported that exosomes containing PD-L1 from non-small cell lung cancer (NSCLC) cells promoted cell stemness [9].

### Reduction of oxidative stress and inflammation

Oxidative stress plays a pivotal role in the performance of TSPCs. Metabolic activities produce reactive oxygen species (ROS), which can cause cellular harm, resulting in early injury and the deterioration of TSPCs functionality. Oxidative stress can upset the equilibrium between self-renewal and differentiation in TSPCs, fostering a phenotype associated with rupture. Inflammation is another critical factor that influences TSPCs function. Pro-inflammatory cytokines, such as interleukin-1(IL-1) and tumor necrosis factor-alpha(TNF-α), can induce cellular functional decline in TSPCs through multiple mechanisms. Cytokines activate the NF-κB pathway, upregulating genes and inflammatory mediators, thereby worsening the inflammatory phenotype [75]. Chronic inflammation, often associated with tendon injuries and diseases, can contribute to the depletion and dysfunction of the TSPCs pool, impairing the regenerative capacity of tendons.

Exosomes have emerged as a potent tool in alleviating the adverse impacts of oxidative stress and inflammation in TSPCs. They achieve this by bolstering antioxidant defense mechanisms and modulating inflammatory pathways [88]. Specifically, Exosomes convey essential antioxidant enzymes, including superoxide dismutase (SOD) and catalase, to TSPCs. This transfer enhances the cells capacity to neutralize ROS, thereby diminishing oxidative damage [89]. Furthermore, exosomes exhibit the ability to suppress the expression of pro-inflammatory cytokines, chemokines, and matrix metalloproteinases in TSPCs [90]. By dampening inflammation, exosomes foster a microenvironment conducive to tendon repair, highlighting their therapeutic potential in regenerative medicine.

### Epigenetic regulation

Epigenetic modifications play a crucial role in the functionality of TSPCs, with changes in the epigenome including DNA methylation, histone modifications, and chromatin reorganization being key factors [91, 92]. These alterations can disrupt gene expression, thus affecting the delicate equilibrium between self-renewal and differentiation capabilities in TSPCs. Consequently, epigenetic changes—encompassing DNA methylation, histone modifications, and chromatin remodeling—significantly contribute to the development of the functional phenotype in TSPCs [93].

Exosomes have been reported to modulate the epigenetic landscape of TSPCs, potentially through the transfer of epigenetic modulators, such as miRNAs, long non-coding RNAs (lncRNAs), and chromatin-remodeling enzymes [94]. For instance, exosomes derived from stem cells have been found to upregulate the expression of miRNAs involved in the regulation of cellular function, such as miR-335 [95]. These miRNAs can target key genes and pathways, thereby promoting the reversal of the cellular functional phenotype in TSPCs.

Stem cell-derived exosomes show significant potential in mitigating the cellular functional phenotype of TSPCs and enhancing their regenerative capacity, offering a promising approach for treating tendon degeneration and TBI injury. By targeting multiple underlying mechanisms, such as signaling pathways, telomere dynamics, mitochondrial function, stemness maintenance, oxidative stress, inflammation, and epigenetic regulation, exosomes can potentially restore the functional and regenerative potential of TSPCs.

## Discussion

The results indicate that exosomes effectively influence TSPCs function through the following therapeutic mechanisms: 1. Modulation of senescence-associated signaling Pathways. 2. Restoration of telomere length and telomerase activity. 3. Improvement of mitochondrial function. 4. Promotion of stemness maintenance. 5. Reduction of oxidative stress and inflammation. 6. Epigenetic regulation. The forthcoming sections will delve into two key areas: Current research.and comparison with other therapeutic strategies. 

### Current research

Bi et al. first identified TSPCs as multipotent cells in human and mouse tendon tissues [8, 96]. TSPCs display typical stem cell markers and possess genes specific to the tendon lineage. Antecedent explorations have demonstrated a significant link between functional changes in TSPCs and TBI injuries [66]. Reinvigorated TSPCs demonstrate enhanced self-renewal, migratory abilities, and tenogenic differentiation potential as opposed to their less mature equivalents [97].

MSCs are gaining more attention in the field of regenerative medicine due to their capacity to differentiate into various cell lineages and their potential to modulate immune responses [98]. The therapeutic use of MSC-derived exosomes is a burgeoning field. Xu et al. described a strategy enhancing MSC-EVs with bioactive glasses, which increased levels of angiogenesis-promoting miR-199b-3p and miR-125a-5p [99]. By loading exosomes with specific miRNAs, such as miR-29a-3p, they can target healing pathways, as shown by Yao et al., who demonstrated the potential of HUMSC-Exos in tendon repair via the PTEN/mTOR/TGF-β1 pathway [100, 101]. MSC-derived exosomes replicate the benefits of their parent cells with the added advantages of reduced immune rejection and tumor risk compared to direct cell therapies [102]. In addition to MSCs, other stem cell sources have been explored for their potential to generate therapeutic exosomes. Exosomes from ADSCs offer a promising therapeutic approach in regenerative medicine, especially for modulating cellular aging in TSPCs. ADSCs have emerged as a valuable source of therapeutic exosomes, as they can be readily harvested and expanded in vitro, and their secretome has been shown to possess potent regenerative and anti-inflammatory properties [103]. ADSCs exosomes impact TSPCs function by delivering specific miRNAs and growth factors. MiRNAs like miR-21 and miR-145 target pathways related to cell function and apoptosis [104]. By modulating these miRNAs, ADSCs exosomes can potentially reduce the debilitated cell population in tendons, thus promoting a more youthful and resilient cellular environment. Beyond tendon-bone healing, stem cell-derived exosomes have demonstrated therapeutic potential in a variety of regenerative and pathological contexts. For instance, the immunomodulatory and tissue regenerative properties of extracellular vesicles may underpin their potential as a therapeutic strategy for COVID-19 [105]. In regenerative medicine research, the presence of various factors, including exosomes, may promote cell proliferation and induce stem cell migration, thereby potentially reducing inflammation and pain and enhancing tissue repair [106]. In conclusion, the exploration of MSCs and ADSCs-derived exosomes marks a significant advancement in the field of regenerative medicine. These exosomes hold the promise of not only revolutionizing tendon repair but also demonstrate the potential of targeted therapies to overcome the limitations of conventional treatments. As research continues to advance, the precise and efficient application of these exosomes may pave the way for innovative and effective interventions in tendon-bone healing and the broader realm of regenerative therapies.

### Comparison with other therapeutic strategies

Surgical interventions, including tendon repair or grafting, provide a more direct approach to restoring tendon function [107]. These methods can be effective for severe tendon injuries or degenerative conditions but carry risks such as infection, prolonged recovery times, and the potential for non-ideal outcomes like scar tissue formation, which can further impair tendon function [108]. Furthermore, surgeries do not specifically target cells, failing to prevent future degeneration caused by cellular function decline. Pharmacological treatments that involve the administration of anti-inflammatory drugs or supplements aimed at reducing symptoms and improving tissue health are another common strategy [109]. While these can alleviate pain and inflammation associated with TBI injuries, they do not restore the characteristics of tendon cells and may not halt the progression of cellular function. Regenerative medicine, especially stem cell and gene therapies, presents promising alternatives. Stem cell therapies involve the transplantation of healthy cells into damaged tissues to restore cellular function [26]. However, the integration and longevity of these cells can be unpredictable, and the risk of immune rejection or abnormal differentiation remains a significant challenge. Gene therapy, which targets specific genes involved in inflammational and degenerative processes, holds potential for directly reversing or mitigating cellular function in TSPCs [110]. This approach, however, is still in its early stages and faces numerous technical, ethical, and regulatory hurdles before it can become a mainstream treatment option. Exosome therapy, with its distinct advantages, is considered a powerful adjunct to traditional treatment methods, particularly in targeting cellular injury.

Exosome therapy, which utilizes exosomes sourced from young and healthy cells, presents a novel approach to targeting the cellular and molecular pathways that contribute to injuries. These exosomes are capable of transporting specific proteins, lipids, and nucleic acids, which can potentially rejuvenate TSPCs, reversing their state of cellular damage and regaining their regenerative potential [28]. Compared to cell therapies, exosomes are less prone to immune rejection due to their lower immunogenicity, making them an appealing option for long-term treatments and for individuals with weakened health. Furthermore, exosomes can be genetically engineered to carry specific therapeutic payloads, which enhances their efficacy and precision compared to conventional drug and regenerative therapies [27]. They also provide a more scalable and less invasive alternative to surgical procedures. The capacity to administer exosomes repeatedly and through minimally invasive means offers a practical advantage over more invasive treatment strategies.

In summary, while each therapeutic strategy for managing tendon injury has its strengths, exosome therapy stands out for its potential to directly alter the cellular environment of TSPCs [31]. By addressing the underlying causes of cellular functional decline and providing a platform for targeted, bio-specific intervention, exosomes represent a promising frontier in regenerative medicine that could surpass the limitations of current treatments in terms of efficacy, safety, and patient compliance. Further research and clinical trials are crucial for validating the efficacy and optimizing the delivery mechanisms of exosome therapy, potentially establishing it as a key treatment for TBI injuries.

## Challenges and translational prospects

While stem cell-derived exosomes hold significant promise in influencing the function of TSPCs and enhancing tendon-bone healing, several challenges and difficulties remain. Firstly, the clinical indications for exosome therapy are not yet well-defined, and the sources of parent stem cells for exosome extraction are limited [36]. Secondly, the challenges of scaling up exosome production, complying with Good Manufacturing Practice standards, and regulatory frameworks must be addressed [111]. Additionally, the swift elimination of exosomes within the body might restrict their enduring therapeutic impact, and the variability of exosomes stemming from diverse cultivation settings and cell generations presents additional hurdles. To surmount these impediments, it is necessary to standardize exosome isolation protocols and employ consistent methods for exosome isolation to reduce variability in size, composition, and function. Furthermore, research into long-term preservation methods for exosomes is needed to extend their shelf life.

### Prospects for clinical translation

Currently, there are at least 150 clinical trials registered on platforms like Clinicaltrials.gov that involve exosomes, with indications ranging from respiratory diseases to infectious diseases, diabetes, and cancer [112]. Among these, 31 utilize stem cell-derived exosomes, primarily from MSCs sourced from various tissues. These clinical trials underscore the growing interest in exosome-based therapies, which may partially replicate the therapeutic effects of their donor cells while avoiding some of the pitfalls associated with live cell transplantation, such as immune reactions and potential tumorigenic risks [16]. Moreover, stem cell-derived exosomes are easier to store long-term. However, the stability and mechanisms of action of these exosomes require further investigation. Administration routes for exosomes are diverse, with intravenous injection being the most common, followed by intraperitoneal injection, intralesional injection, oral administration, and nasal spray. Notably, engineered exosomes like exoSTING8, which is being trialed by Codiak Biosciences for solid tumors, represent the future direction of stem cell exosome therapy.

Exosomes’ potential to change the function of TSPCs offers significant promise for developing cell-free, exosome-based therapies for tendon disorders. Current research aims to optimize the isolation, characterization, and therapeutic delivery of exosomes to improve their clinical efficacy [4]. Additionally, the identification of specific exosomal cargoes and their underlying mechanisms of action in the context of tendon regeneration will further inform the design of targeted exosome-based interventions [88]. Integrating exosome-mediated TSPCs rejuvenation with other regenerative strategies, such as biomaterial scaffolds and growth factor supplementation, may synergistically improve tendon repair and regeneration, ultimately leading to better clinical outcomes for patients with tendon-related pathologies.

### Future research directions

Future research in exosome therapy for TBI injuries should focus on harnessing exosomes’ regenerative capabilities to develop more effective tendon repair and rejuvenation treatments. This includes identifying the most potent molecular components within exosomes to revitalize tendon cells, enhancing exosome regenerative properties through genetic engineering or synthetic nanoparticles, and discovering new biomarkers and signaling pathways to guide targeted exosome therapies [28]. Additionally, gene editing in exosome donor cells could lead to the creation of ‘designer exosomes’ tailored for specific therapeutic outcomes [27]. As research progresses, establishing robust frameworks for patient consent, treatment protocols, and long-term monitoring will be essential, along with the development of international standards and regulations to ensure the safety, efficacy, and accessibility of these therapies. The future of exosome therapy research is broad, offering unique challenges and opportunities, with interdisciplinary collaboration being key to realizing the full therapeutic potential of exosomes in tendon repair and regeneration.

## Conclusion

Stem cell-derived exosomes have emerged as a potent therapeutic strategy, effectively targeting the mechanisms underlying cellular function of TSPCs and thereby enhancing tendon-bone healing. These exosomes rejuvenate and reinvigorate critical cell populations, which is pivotal for tendon repair and regeneration. As ongoing research continues to explore their potential, it holds promise for uncovering new strategies to cure TBI injuries and improve tendon-bone healing.

## Data Availability

No datasets were generated or analysed during the current study.

## References

[1] Zhang M, Liu H, Cui Q, Han P, Yang S, Shi M, et al. Tendon stem cell-derived exosomes regulate inflammation and promote the high-quality healing of injured tendon. Stem Cell Res Ther. 2020;11(1):402. 32943109 10.1186/s13287-020-01918-x PMC7499865

[2] Yang R, Li G, Zhuang C, Yu P, Ye T, Zhang Y et al. Gradient bimetallic ion-based hydrogels for tissue microstructure reconstruction of tendon-to-bone insertion. Sci Adv. 2021;7(26). 10.1126/sciadv.abg3816 PMC8221628 34162547

[3] Fang WH, Agrawal DK, Thankam FG. Smart exosomes: A smart approach for tendon regeneration. Tissue Eng Part B: Reviews. 2022;28(3):613–25. 10.1089/ten.TEB.2021.0075 34074136

[4] Qin B, Bao D, Liu Y, Zeng S, Deng K, Liu H, et al. Engineered exosomes: a promising strategy for tendon-bone healing. J Adv Res. 2024;64:155–69. 37972886 10.1016/j.jare.2023.11.011 PMC11464473

[5] Zou M, Wang J, Shao Z. Therapeutic potential of exosomes in tendon and tendon-Bone healing: A systematic review of preclinical studies. J Funct Biomater. 2023;14(6). 10.3390/jfb14060299 PMC10299056 37367263

[6] Zhang C, Zhu J, Zhou Y, Thampatty BP, Wang JHC. Tendon stem/progenitor cells and their interactions with extracellular matrix and mechanical loading. Stem Cells Int. 2019;2019:3674647. 31737075 10.1155/2019/3674647 PMC6815631

[7] Migliorini F, Tingart M, Maffulli N. Progress with stem cell therapies for tendon tissue regeneration. Expert Opin Biol Ther. 2020;20(11):1373–9. 32574078 10.1080/14712598.2020.1786532

[8] Bi Y, Ehirchiou D, Kilts TM, Inkson CA, Embree MC, Sonoyama W, et al. Identification of tendon stem/progenitor cells and the role of the extracellular matrix in their niche. Nat Med. 2007;13(10):1219–27. 17828274 10.1038/nm1630

[9] Hong W, Xue M, Jiang J, Zhang Y, Gao X. Circular RNA circ-CPA4/ let-7 miRNA/PD-L1 axis regulates cell growth, stemness, drug resistance and immune evasion in non-small cell lung cancer (NSCLC). J Exp Clin Cancer Res. 2020;39(1):149. 32746878 10.1186/s13046-020-01648-1 PMC7397626

[10] TROWBRIDGE CRHaIS. Internalization and processing of transferrin and the transferrin receptor in human carcinoma A431 cells. J Cell Biol. 1983. 10.1083/jcb.97.2.508 PMC2112524 6309862

[11] Johnstone B-TPRM. Fate of the transferrin receptor during maturation of sheep reticulocytes in vitro. Selective Externalization of the Receptor Cell; 1983. 10.1016/0092-8674(83)90040-5 6307529

[12] Mashouri L, Yousefi H, Aref AR, Ahadi AM, Molaei F, Alahari SK. Exosomes: composition, biogenesis, and mechanisms in cancer metastasis and drug resistance. Mol Cancer. 2019;18(1):75. 30940145 10.1186/s12943-019-0991-5 PMC6444571

[13] Wu KY, Ahmad H, Lin G, Carbonneau M, Tran SD. Mesenchymal stem Cell-Derived exosomes in ophthalmology: A comprehensive review. Pharmaceutics. 2023;15(4). 10.3390/pharmaceutics15041167 PMC10142951 37111652

[14] Lei J, Jiang X, Li W, Ren J, Wang D, Ji Z, et al. Exosomes from antler stem cells alleviate mesenchymal stem cell senescence and osteoarthritis. Protein Cell. 2021;13(3):220–6. 34342820 10.1007/s13238-021-00860-9 PMC8901817

[15] Gao M, Yu Z, Yao D, Qian Y, Wang Q, Jia R. Mesenchymal stem cells therapy: A promising method for the treatment of uterine scars and premature ovarian failure. Tissue Cell. 2022;74:101676. 34798583 10.1016/j.tice.2021.101676

[16] Jiang L, Lu J, Chen Y, Lyu K, Long L, Wang X, et al. Mesenchymal stem cells: an efficient cell therapy for tendon repair (Review). Int J Mol Med. 2023;52(2):1. 10.3892/ijmm.2023.5273 PMC10373123 37387410

[17] Pegtel DM, Gould SJ, Exosomes. Annu Rev Biochem. 2019;88:487–514. 31220978 10.1146/annurev-biochem-013118-111902

[18] Jin S, Wang Y, Wu X, Li Z, Zhu L, Niu Y, et al. Young exosome Bio-Nanoparticles restore Aging-Impaired tendon stem/progenitor cell function and reparative capacity. Adv Mater. 2023;35(18):e2211602. 36779444 10.1002/adma.202211602

[19] Lanci A, Merlo B, Grandis A, Mariella J, Castagnetti C, Iacono E. Gross and histological examination of wharton’s jelly in the equine umbilical cord. Theriogenology. 2023;209:184–92. 37421877 10.1016/j.theriogenology.2023.06.032

[20] Hu N, Cai Z, Jiang X, Wang C, Tang T, Xu T, et al. Hypoxia-pretreated ADSC-derived exosome-embedded hydrogels promote angiogenesis and accelerate diabetic wound healing. Acta Biomater. 2023;157:175–86. 36503078 10.1016/j.actbio.2022.11.057

[21] Salhotra A, Shah HN, Levi B, Longaker MT. Mechanisms of bone development and repair. Nat Rev Mol Cell Biol. 2020;21(11):696–711. 32901139 10.1038/s41580-020-00279-w PMC7699981

[22] Hjorthaug GA, Søreide E, Nordsletten L, Madsen JE, Reinholt FP, Niratisairak S, et al. Negative effect of Zoledronic acid on tendon-to-bone healing. Acta Orthop. 2018;89(3):360–6. 29493345 10.1080/17453674.2018.1440189 PMC6055777

[23] Chen Y, Wu Y, Guo L, Yuan S, Sun J, Zhao K, et al. Exosomal Lnc NEAT1 from endothelial cells promote bone regeneration by regulating macrophage polarization via DDX3X/NLRP3 axis. J Nanobiotechnol. 2023;21(1):98. 10.1186/s12951-023-01855-w PMC10029245 36941678

[24] Liu W, Yu M, Chen F, Wang L, Ye C, Chen Q, et al. A novel delivery nanobiotechnology: engineered miR-181b exosomes improved osteointegration by regulating macrophage polarization. J Nanobiotechnol. 2021;19(1):269. 10.1186/s12951-021-01015-y PMC8424816 34493305

[25] Lu G-d, Cheng P, Liu T, Wang Z. BMSC-Derived Exosomal miR-29a promotes angiogenesis and osteogenesis. Front Cell Dev Biology. 2020;8:608521. 10.3389/fcell.2020.608521 PMC7755650 33363169

[26] Zou J, Yang W, Cui W, Li C, Ma C, Ji X, et al. Therapeutic potential and mechanisms of mesenchymal stem cell-derived exosomes as bioactive materials in tendon-bone healing. J Nanobiotechnol. 2023;21(1):14. 10.1186/s12951-023-01778-6 PMC9841717 36642728

[27] Chen D, Tang Q, Song W, He Y. Platelet-derived exosomes alleviate tendon stem/progenitor cell senescence and ferroptosis by regulating AMPK/Nrf2/GPX4 signaling and improve tendon-bone junction regeneration in rats. J Orthop Surg Res. 2024;19(1):382. 38943181 10.1186/s13018-024-04869-8 PMC11212425

[28] Lu J, Yang X, He C, Chen Y, Li C, Li S, et al. Rejuvenation of tendon stem/progenitor cells for functional tendon regeneration through platelet-derived exosomes loaded with Recombinant Yap1. Acta Biomater. 2023;161:80–99. 36804538 10.1016/j.actbio.2023.02.018

[29] Yin H, Strunz F, Yan Z, Lu J, Brochhausen C, Kiderlen S, et al. Three-dimensional self-assembling nanofiber matrix rejuvenates aged/degenerative human tendon stem/progenitor cells. Biomaterials. 2020;236:119802. 32014804 10.1016/j.biomaterials.2020.119802

[30] Sunwoo JY, Eliasberg CD, Carballo CB, Rodeo SA. The role of the macrophage in tendinopathy and tendon healing. J Orthop Res. 2020;38(8):1666–75. 32190920 10.1002/jor.24667

[31] Zhang X, Song W, Liu Y, Han K, Wu Y, Cho E, et al. Healthy tendon stem Cell-Derived exosomes promote tendon-To-Bone healing of aged chronic rotator cuff tears by breaking the Positive-Feedback Cross-Talk between senescent tendon stem cells and macrophages through the modulation of macrophage polarization. Small. 2024;20(31):e2311033. 38459643 10.1002/smll.202311033

[32] Li Z, Li Q, Tong K, Zhu J, Wang H, Chen B, et al. BMSC-derived exosomes promote tendon-bone healing after anterior cruciate ligament reconstruction by regulating M1/M2 macrophage polarization in rats. Stem Cell Res Ther. 2022;13(1):295. 35841008 10.1186/s13287-022-02975-0 PMC9284827

[33] Huang Y, He B, Wang L, Yuan B, Shu H, Zhang F, et al. Bone marrow mesenchymal stem cell-derived exosomes promote rotator cuff tendon-bone healing by promoting angiogenesis and regulating M1 macrophages in rats. Stem Cell Res Ther. 2020;11(1):496. 33239091 10.1186/s13287-020-02005-x PMC7687785

[34] Zhu Y, Yan J, Zhang H, Cui G. Bone marrow mesenchymal stem cell–derived exosomes: A novel therapeutic agent for tendon–bone healing (Review). Int J Mol Med. 2023;52(6). 10.3892/ijmm.2023.5324 PMC10635703 37937691

[35] Chen W, Sun Y, Gu X, Cai J, Liu X, Zhang X, et al. Conditioned medium of human bone marrow-derived stem cells promotes tendon-bone healing of the rotator cuff in a rat model. Biomaterials. 2021;271:120714. 33610048 10.1016/j.biomaterials.2021.120714

[36] Yang C, Teng Y, Geng B, Xiao H, Chen C, Chen R, et al. Strategies for promoting tendon-bone healing: current status and prospects. Front Bioeng Biotechnol. 2023;11:1118468. 36777256 10.3389/fbioe.2023.1118468 PMC9911882

[37] Xu J, Ye Z, Han K, Zheng T, Zhang T, Dong S, et al. Infrapatellar fat pad mesenchymal stromal Cell-Derived exosomes accelerate Tendon-Bone healing and Intra-articular graft remodeling after anterior cruciate ligament reconstruction. Am J Sports Med. 2022;50(3):662–73. 35224997 10.1177/03635465211072227

[38] Rong X, Tang Y, Cao S, Xiao S, Wang H, Zhu B, et al. An extracellular Vesicle-Cloaked multifaceted biocatalyst for Ultrasound-Augmented tendon matrix reconstruction and immune microenvironment regulation. ACS Nano. 2023;17(17):16501–16. 37616178 10.1021/acsnano.3c00911

[39] Ren Y, Zhang S, Wang Y, Jacobson DS, Reisdorf RL, Kuroiwa T, et al. Effects of purified exosome product on rotator cuff tendon-bone healing in vitro and in vivo. Biomaterials. 2021;276:121019. 34325337 10.1016/j.biomaterials.2021.121019 PMC9707649

[40] Li J, Liu Z-P, Xu C, Guo A. TGF-beta1-containing exosomes derived from bone marrow mesenchymal stem cells promote proliferation, migration and fibrotic activity in rotator cuff tenocytes. Regen Ther. 2020;15:70–6. 33426204 10.1016/j.reth.2020.07.001 PMC7770343

[41] Song K, Jiang T, Pan P, Yao Y, Jiang Q. Exosomes from tendon derived stem cells promote tendon repair through miR-144-3p-regulated tenocyte proliferation and migration. Stem Cell Res Ther. 2022;13(1):80. 35197108 10.1186/s13287-022-02723-4 PMC8867681

[42] Yu H, Cheng J, Shi W, Ren B, Zhao F, Shi Y, et al. Bone marrow mesenchymal stem cell-derived exosomes promote tendon regeneration by facilitating the proliferation and migration of endogenous tendon stem/progenitor cells. Acta Biomater. 2020;106:328–41. 32027991 10.1016/j.actbio.2020.01.051

[43] Gu J, Zhang Q, Geng M, Wang W, Yang J, Khan AUR, et al. Construction of nanofibrous scaffolds with interconnected perfusable microchannel networks for engineering of vascularized bone tissue. Bioact Mater. 2021;6(10):3254–68. 33778203 10.1016/j.bioactmat.2021.02.033 PMC7970223

[44] Baldino L, Cardea S, Maffulli N, Reverchon E. Regeneration techniques for bone-to-tendon and muscle-to-tendon interfaces reconstruction. Br Med Bull. 2016;117(1):25–37. 26837850 10.1093/bmb/ldv056

[45] Connor DE, Paulus JA, Dabestani PJ, Thankam FK, Dilisio MF, Gross RM, et al. Therapeutic potential of exosomes in rotator cuff tendon healing. J Bone Miner Metab. 2019;37(5):759–67. 31154535 10.1007/s00774-019-01013-z PMC6830879

[46] Wang W, Liang X, Zheng K, Ge G, Chen X, Xu Y, et al. Horizon of exosome-mediated bone tissue regeneration: the all-rounder role in biomaterial engineering. Mater Today Bio. 2022;16:100355. 35875196 10.1016/j.mtbio.2022.100355 PMC9304878

[47] Lai F, Wang J, Tang H, Huang P, Liu J, He G, et al. VEGF promotes tendon regeneration of aged rats by inhibiting adipogenic differentiation of tendon stem/progenitor cells and promoting vascularization. FASEB J. 2022;36(8):e22433. 35867348 10.1096/fj.202200213R

[48] Komorowski J, Jerczynska H, Siejka A, Baranska P, Lawnicka H, Pawlowska Z, et al. Effect of thalidomide affecting VEGF secretion, cell migration, adhesion and capillary tube formation of human endothelial ea.hy 926 cells. Life Sci. 2006;78(22):2558–63. 16310808 10.1016/j.lfs.2005.10.016

[49] Wu D, Chang X, Tian J, Kang L, Wu Y, Liu J, et al. Bone mesenchymal stem cells stimulation by magnetic nanoparticles and a static magnetic field: release of Exosomal miR-1260a improves osteogenesis and angiogenesis. J Nanobiotechnol. 2021;19(1):209. 10.1186/s12951-021-00958-6 PMC8278669 34256779

[50] Liu W, Li L, Rong Y, Qian D, Chen J, Zhou Z, et al. Hypoxic mesenchymal stem cell-derived exosomes promote bone fracture healing by the transfer of miR-126. Acta Biomater. 2020;103:196–212. 31857259 10.1016/j.actbio.2019.12.020

[51] Zhang T, Yan S, Song Y, Chen C, Xu D, Lu B, et al. Exosomes secreted by hypoxia-stimulated bone-marrow mesenchymal stem cells promote grafted tendon-bone tunnel healing in rat anterior cruciate ligament reconstruction model. J Orthop Translat. 2022;36:152–63. 36263381 10.1016/j.jot.2022.08.001 PMC9550857

[52] Wang C, Hu Q, Song W, Yu W, He Y. Adipose stem Cell-Derived exosomes decrease fatty infiltration and enhance rotator cuff healing in a rabbit model of chronic tears. Am J Sports Med. 2020;48(6):1456–64. 32272021 10.1177/0363546520908847

[53] Plummer J, Park M, Perodin F, Horowitz MC, Hens JR. Methionine-Restricted diet increases MiRNAs that can target RUNX2 expression and alters bone structure in young mice. J Cell Biochem. 2017;118(1):31–42. 27191548 10.1002/jcb.25604 PMC5426510

[54] Lin Z, He H, Wang M, Liang J. MicroRNA-130a controls bone marrow mesenchymal stem cell differentiation towards the osteoblastic and adipogenic fate. Cell Prolif. 2019;52(6):e12688. 31557368 10.1111/cpr.12688 PMC6869834

[55] Zhong Y, Li X, Wang F, Wang S, Wang X, Tian X, et al. Emerging potential of exosomes on adipogenic differentiation of mesenchymal stem cells. Front Cell Dev Biology. 2021;9:649552. 10.3389/fcell.2021.649552 PMC8258133 34239869

[56] Phalke S, Mzoughi S, Bezzi M, Jennifer N, Mok WC, Low DHP, et al. p53-Independent regulation of p21Waf1/Cip1 expression and senescence by PRMT6. Nucleic Acids Res. 2012;40(19):9534–42. 22987071 10.1093/nar/gks858 PMC3479215

[57] Li S, Sun Y, Chen Y, Lu J, Jiang G, Yu K, et al. Sandwich biomimetic scaffold based tendon stem/progenitor cell alignment in a 3D microenvironment for functional tendon regeneration. ACS Appl Mater Interfaces. 2023;15(3):4652–67. 36698266 10.1021/acsami.2c16584

[58] Takahashi A, Ohtani N, Yamakoshi K, Iida S-i, Tahara H, Nakayama K, et al. Mitogenic signalling and the p16INK4a-Rb pathway cooperate to enforce irreversible cellular senescence. Nat Cell Biol. 2006;8(11):1291–7. 17028578 10.1038/ncb1491

[59] He L, Zhu C, Jia J, Hao X-Y, Yu X-Y, Liu X-Y et al. ADSC-Exos containing MALAT1 promotes wound healing by targeting miR-124 through activating Wnt/beta-catenin pathway. Biosci Rep. 2020;40(5). 10.1042/BSR20192549 PMC7214401 32342982

[60] Chen M, Li Y, Xiao L, Dai G, Lu P, Rui Y. Noncanonical Wnt5a signaling regulates tendon stem/progenitor cells senescence. Stem Cell Res Ther. 2021;12(1):544. 34663475 10.1186/s13287-021-02605-1 PMC8521898

[61] Alberton P, Dex S, Popov C, Shukunami C, Schieker M, Docheva D. Loss of Tenomodulin results in reduced Self-Renewal and augmented senescence of tendon stem/progenitor cells. Stem Cells Dev. 2015;24(5):597–609. 25351164 10.1089/scd.2014.0314 PMC4333258

[62] Chen M, Xiao L, Dai G, Lu P, Zhang Y, Li Y, et al. Inhibition of JAK-STAT signaling pathway alleviates Age-Related phenotypes in tendon stem/progenitor cells. Front Cell Dev Biol. 2021;9:650250. 33855026 10.3389/fcell.2021.650250 PMC8039155

[63] Chen D, Tang Q, Song W, He Y. Platelet-derived exosomes alleviate tendon stem/progenitor cell senescence and ferroptosis by regulating AMPK/Nrf2/GPX4 signaling and improve tendon-bone junction regeneration in rats. J Orthop Surg Res. 2024;19(1). 10.1186/s13018-024-04869-8 PMC11212425 38943181

[64] Han W, Gu D, Li X, Chen H, Tao X, Chen L. Young TSPC-Derived Exosomal circPVT1 ameliorates Aging-Impaired cell function via SIRT1/NF-κB. Tissue Eng Part C: Methods. 2024;30(6):248–54. 38842177 10.1089/ten.TEC.2024.0057

[65] Rui Y-f, Chen M-h, Li Y-j, Geng XL-f, Wang P et al. P,. CTGF Attenuates Tendon-Derived Stem/Progenitor Cell Aging. Stem Cells International. 2019;2019(2019):1–12. 10.1155/2019/6257537 PMC6881574 31827530

[66] Chen M, Li Y, Xiao L, Dai G, Lu P, Wang Y, et al. AQP1 modulates tendon stem/progenitor cells senescence during tendon aging. Cell Death Dis. 2020;11(3):193. 32188840 10.1038/s41419-020-2386-3 PMC7080760

[67] Wang Y, Jin S, Luo D, He D, Yu M, Zhu L, et al. Prim-O-glucosylcimifugin ameliorates aging-impaired endogenous tendon regeneration by rejuvenating senescent tendon stem/progenitor cells. Bone Res. 2023;11(1):54. 37872152 10.1038/s41413-023-00288-3 PMC10593834

[68] Cai Z, Xin Z, Wang H, Wang C, Liu X. Extracellular Vesicle-Contained thrombospondin 1 retards Age‐Related degenerative tendinopathy by rejuvenating tendon stem/progenitor cell senescence. Small. 2024. 10.1002/smll.202400598 38778750

[69] Wang H, Cai Z, Ying M, Song W, Liu X, Wei H, et al. Glutamine promotes rotator cuff healing by ameliorating Age-Related osteoporosis. J Bone Joint Surg. 2025;107(9):948–57. 40146808 10.2106/JBJS.24.00779

[70] Hawthorne BC, Wellington IJ, Sabitsky JT, Murphy KV, Karsmarski OP, Thomas RO, et al. Human rotator cuff tears reveal an Age-Dependent increase in markers of cellular senescence and selective removal of senescent cells with Dasatinib + Quercetin increases genetic expression of COL1A1 in vitro. Arthroscopy: J Arthroscopic Relat Surg. 2024;40(1):34–44. 10.1016/j.arthro.2023.05.036 PMC10746834 37356505

[71] Dai G, Li Y, Zhang M, Lu P, Zhang Y, Wang H, et al. The regulation of the ampk/mtor Axis mitigates tendon stem/progenitor cell senescence and delays tendon aging. Stem Cell Rev Rep. 2023;19(5):1492–506. 36917311 10.1007/s12015-023-10526-0

[72] Han W, Gu D, Chen H, Tao X, Chen L. HPF1 regulates tendon stem/progenitor cell senescence and tendon repair via PARP1-mediated poly-ADP ribosylation of HuR. Genes Genomics. 2024;46(1):27–36. 37713069 10.1007/s13258-023-01447-w

[73] Cai Z, Zhang Y, Liu S, Liu X, Celecoxib. Beyond Anti-inflammation, alleviates Tendon-Derived stem cell senescence in degenerative rotator cuff tendinopathy. Am J Sports Med. 2022;50(9):2488–96. 35666137 10.1177/03635465221098133

[74] Nie D, Zhang J, Zhou Y, Sun J, Wang W, Wang JHC. Rapamycin treatment of tendon stem/progenitor cells reduces cellular senescence by upregulating autophagy. Stem Cells Int. 2021;2021:1–10. 10.1155/2021/6638249 PMC7870298 33603790

[75] Wang C, Zhou Z, Song W, Cai Z, Ding Z, Chen D, et al. Inhibition of IKKbeta/NF-kappaB signaling facilitates tendinopathy healing by rejuvenating inflamm-aging induced tendon-derived stem/progenitor cell senescence. Mol Ther Nucleic Acids. 2022;27:562–76. 35036066 10.1016/j.omtn.2021.12.026 PMC8738957

[76] Anoop M, Datta I. Stem cells derived from human exfoliated deciduous teeth (SHED) in neuronal disorders: A review. Curr Stem Cell Res Ther. 2021;16(5):535–50. 33349220 10.2174/1574888X16666201221151512

[77] Han W, Tao X, Weng T, Chen L, Circular. RNA PVT1 inhibits tendon stem/progenitor cell senescence by sponging microRNA-199a-5p. Toxicol Vitro. 2022;79:105297. 10.1016/j.tiv.2021.105297 34896603

[78] Chen P, Chen Z, Mitchell C, Gao J, Chen L, Wang A, et al. Intramuscular injection of botox causes tendon atrophy by induction of senescence of tendon-derived stem cells. Stem Cell Res Ther. 2021;12(1):38. 33413592 10.1186/s13287-020-02084-w PMC7791643

[79] De Bernardes B, Blasco MA. Telomerase at the intersection of cancer and aging. Trends Genet. 2013;29(9):513–20. 23876621 10.1016/j.tig.2013.06.007 PMC3896987

[80] Ju Z, Rudolph L. Telomere dysfunction and stem cell ageing. Biochimie. 2008;90(1):24–32. 18029082 10.1016/j.biochi.2007.09.006

[81] Ogawa M, Udono M, Teruya K, Uehara N, Katakura Y. Exosomes derived from Fisetin-Treated keratinocytes mediate hair growth promotion. Nutrients. 2021;13(6):2087. 34207142 10.3390/nu13062087 PMC8234638

[82] Burlacu C-C, Ciobanu D, Badulescu A-V, Chelaru V-F, Mitre A-O, Capitanescu B et al. Circulating MicroRNAs and extracellular Vesicle-Derived MicroRNAs as predictors of functional recovery in ischemic stroke patients: A systematic review and Meta-Analysis. Int J Mol Sci. 2022;24(1). 10.3390/ijms24010251 PMC9820088 36613694

[83] Wei B, Ji M, Lin Y, Wang S, Liu Y, Geng R et al. Mitochondrial transfer from bone mesenchymal stem cells protects against tendinopathy both in vitro and in vivo. Stem Cell Res Ther. 2023;14(1). 10.1186/s13287-023-03329-0 PMC10134653 37101277

[84] Wang S, Yao Z, Zhang X, Li J, Huang C, Ouyang Y et al. Energy-Supporting Enzyme‐Mimic nanoscaffold facilitates tendon regeneration based on a mitochondrial protection and microenvironment remodeling strategy. Adv Sci. 2022;9(31). 10.1002/advs.202202542 PMC9631092 36000796

[85] Sharma A. Mitochondrial cargo export in exosomes: possible pathways and implication in disease biology. J Cell Physiol. 2023;238(4):687–97. 36745675 10.1002/jcp.30967

[86] Sun Y, Chen H, Ye H, Liang W, Lam K-k, Cheng B, et al. Nudt21-mediated alternative polyadenylation of HMGA2 3′-UTR impairs stemness of human tendon stem cell. Aging. 2020;12(18):18436–52. 32979259 10.18632/aging.103771 PMC7585117

[87] Fatima F, Nawaz M. Stem cell-derived exosomes: roles in stromal remodeling, tumor progression, and cancer immunotherapy. Chin J Cancer. 2015;34(12):541–53. 26369565 10.1186/s40880-015-0051-5 PMC4593342

[88] He Y, Lu S, Chen W, Yang L, Li F, Zhou P, et al. Exosomes derived from tendon stem/progenitor cells enhance tendon-bone interface healing after rotator cuff repair in a rat model. Bioactive Mater. 2024;40:484–502. 10.1016/j.bioactmat.2024.06.014 PMC11260958 39040569

[89] Liu A, Wang Q, Zhao Z, Wu R, Wang M, Li J, et al. Nitric oxide nanomotor driving Exosomes-Loaded microneedles for Achilles tendinopathy healing. ACS Nano. 2021;15(8):13339–50. 34324304 10.1021/acsnano.1c03177

[90] Jiang L, Lu J, Chen Y, Lyu K, Long L, Wang X et al. Mesenchymal stem cells: an efficient cell therapy for tendon repair (Review). Int J Mol Med. 2023;52(2). 10.3892/ijmm.2023.5273 PMC10373123 37387410

[91] Riasat K, Bardell D, Goljanek-Whysall K, Clegg PD, Peffers MJ. Epigenetic mechanisms in tendon ageing. Br Med Bull. 2020;135(1):90–107. 32827252 10.1093/bmb/ldaa023 PMC7585832

[92] Tarnowski M, Tomasiak P, Tkacz M, Zgutka K, Piotrowska K. Epigenetic alterations in Sports-Related injuries. Genes. 2022;13(8). 10.3390/genes13081471 PMC9408207 36011382

[93] Zong Y, Huang J, Sankarasharma D, Morikawa T, Fukayama M, Epstein JI et al. Stromal epigenetic dysregulation is sufficient to initiate mouse prostate cancer via paracrine Wnt signaling. Proceedings of the National Academy of Sciences. 2012;109(50):E3395-404. 10.1073/pnas.1217982109 PMC3528570 23184966

[94] Luo D, Zhu H, Li S, Wang Z, Xiao J. Mesenchymal stem cell-derived exosomes as a promising cell-free therapy for knee osteoarthritis. Front Bioeng Biotechnol. 2024;12:1309946. 38292826 10.3389/fbioe.2024.1309946 PMC10824863

[95] Tome M, Sepulveda JC, Delgado M, Andrades JA, Campisi J, Gonzalez MA, et al. miR-335 correlates with senescence/aging in human mesenchymal stem cells and inhibits their therapeutic actions through Inhibition of AP-1 activity. Stem Cells. 2014;32(8):2229–44. 24648336 10.1002/stem.1699 PMC4207125

[96] Chen L, Wang G-D, Liu J-P, Wang H-S, Liu X-M, Wang Q, et al. miR-135a modulates tendon stem/progenitor cell senescence via suppressing ROCK1. Bone. 2015;71:210–6. 25460182 10.1016/j.bone.2014.11.001

[97] Zhou Z, Akinbiyi T, Xu L, Ramcharan M, Leong DJ, Ros SJ, et al. Tendon-derived stem/progenitor cell aging: defective self‐renewal and altered fate. Aging Cell. 2010;9(5):911–5. 20569237 10.1111/j.1474-9726.2010.00598.x PMC2944918

[98] Lotfy A, AboQuella NM, Wang H. Mesenchymal stromal/stem cell (MSC)-derived exosomes in clinical trials. Stem Cell Res Ther. 2023;14(1):66. 37024925 10.1186/s13287-023-03287-7 PMC10079493

[99] Xu H, Zhu Y, Hsiao AW-T, Xu J, Tong W, Chang L, et al. Bioactive glass-elicited stem cell-derived extracellular vesicles regulate M2 macrophage polarization and angiogenesis to improve tendon regeneration and functional recovery. Biomaterials. 2023;294:121998. 36641814 10.1016/j.biomaterials.2023.121998

[100] Yao Z, Li J, Xiong H, Cui H, Ning J, Wang S, et al. MicroRNA engineered umbilical cord stem cell-derived exosomes direct tendon regeneration by mTOR signaling. J Nanobiotechnol. 2021;19(1):169. 10.1186/s12951-021-00906-4 PMC8180131 34090456

[101] Lyu K, Liu X, Liu T, Lu J, Jiang L, Chen Y, et al. MiRNAs contributing to the repair of tendon injury. Cell Tissue Res. 2023;393(2):201–15. 37249708 10.1007/s00441-023-03780-8 PMC10406718

[102] Gissi C, Radeghieri A, Antonetti Lamorgese Passeri C, Gallorini M, Calciano L, Oliva F, et al. Extracellular vesicles from rat-bone-marrow mesenchymal stromal/stem cells improve tendon repair in rat Achilles tendon injury model in dose-dependent manner: A pilot study. PLoS ONE. 2020;15(3):e0229914. 32163452 10.1371/journal.pone.0229914 PMC7067391

[103] Song Y, You Y, Xu X, Lu J, Huang X, Zhang J, et al. Adipose-Derived mesenchymal stem Cell-Derived exosomes biopotentiated extracellular matrix hydrogels accelerate diabetic wound healing and skin regeneration. Adv Sci (Weinh). 2023;10(30):e2304023. 37712174 10.1002/advs.202304023 PMC10602544

[104] Holvoet P. Aging and metabolic reprogramming of Adipose-Derived stem cells affect molecular mechanisms related to cardiovascular diseases. Cells. 2023;12(24). 10.3390/cells12242785 PMC10741778 38132104

[105] Gupta A, Shivaji K, Kadam S, Gupta M, Rodriguez HC, Potty AG, et al. Immunomodulatory extracellular vesicles: an alternative to cell therapy for COVID-19. Expert Opin Biol Ther. 2021;21(12):1551–60. 33886388 10.1080/14712598.2021.1921141

[106] Gupta A, Cady C, Fauser AM, Rodriguez HC, Mistovich RJ, Potty AGR et al. Cell-free stem Cell-Derived extract formulation for regenerative medicine applications. Int J Mol Sci. 2020;21(24). 10.3390/ijms21249364 PMC7763336 33316880

[107] Tang JB, Lalonde D, Harhaus L, Sadek AF, Moriya K, Pan ZJ. Flexor tendon repair: recent changes and current methods. J Hand Surg (European Volume). 2021;47(1):31–9. 10.1177/17531934211053757 34738496

[108] Weinfeld SB. Achilles tendon disorders. Med Clin North Am. 2014;98(2):331–8. 24559878 10.1016/j.mcna.2013.11.005

[109] Liang W, Zhou C, Deng Y, Fu L, Zhao J, Long H et al. The current status of various preclinical therapeutic approaches for tendon repair. Ann Med. 2024;56(1). 10.1080/07853890.2024.2337871 PMC11095292 38738394

[110] Tang JB, Zhou YL, Wu YF, Liu PY, Wang XT. Gene therapy strategies to improve strength and quality of flexor tendon healing. Expert Opin Biol Ther. 2016;16(3):291–301. 26853840 10.1517/14712598.2016.1134479

[111] Mendt M, Kamerkar S, Sugimoto H, McAndrews KM, Wu C-C, Gagea M et al. Generation and testing of clinical-grade exosomes for pancreatic cancer. JCI Insight. 2018;3(8). 10.1172/jci.insight.99263 PMC5931131 29669940

[112] Rezaie J, Feghhi M, Etemadi T. A review on exosomes application in clinical trials: perspective, questions, and challenges. Cell Communication Signal. 2022;20(1). 10.1186/s12964-022-00959-4 PMC9483361 36123730

